# Mathematics Matters or Maybe Not: An Astonishing Independence between Mathematics and the Rate of Learning in General Chemistry

**DOI:** 10.1021/jacsau.4c01126

**Published:** 2025-02-26

**Authors:** Kenneth R. Koedinger, Mark Blaser, Elizabeth A. McLaughlin, Hui Cheng, David J. Yaron

**Affiliations:** † Human Computer Interaction Institute, Carnegie Mellon University, Pittsburgh, Pennsylvania 15213, United States; ‡ The Simon Initiative, Carnegie Mellon University, Pittsburgh, Pennsylvania 15213, United States; § Department of Chemistry, Carnegie Mellon University, Pittsburgh, Pennsylvania 15213, United States

**Keywords:** chemical education research, learning rate, learning curves, deliberate practice, educational data mining, logistic regression growth modeling

## Abstract

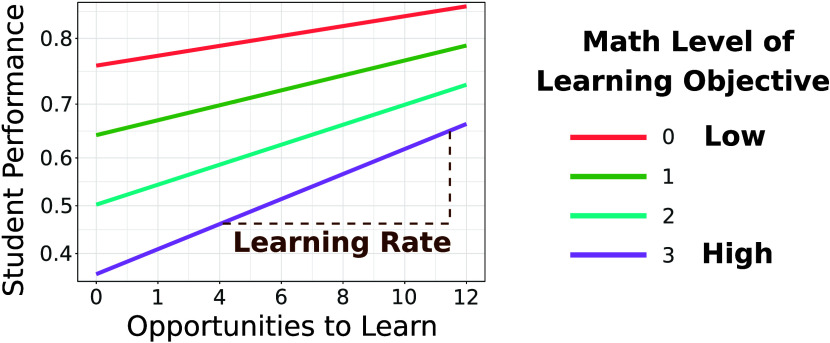

Research spanning nearly a century has found that mathematics plays an important role in the learning of chemistry. Here, we use a large dataset of student interactions with online courseware to investigate the details of this link between mathematics and chemistry. The activities in the courseware are labeled against a list of knowledge components (KCs) covered by the content, and student interactions are tracked over a full semester of general chemistry at a range of institutions. Logistic regression is used to model student performance as a function of the number of opportunities a student has taken to engage with a particular KC. This regression analysis generates estimates of both the initial knowledge and the learning rate for each student and each KC. Consistent with results from other domains, the initial knowledge varies substantially across students, but the learning rate is nearly the same for all students. The role of mathematics is investigated by labeling each KC with the level of math involved. The overwhelming result from regressions based on these labels is that only the initial knowledge varies strongly across students and across the level of math involved in a particular topic. The student learning rate is nearly independent of both the level of math involved in a KC and the prior mathematical preparation of an individual student. The observation that the primary challenge for students lies in initial knowledge, rather than learning rate, may have implications for course and curriculum design.

## 1 Introduction

This work explores how large datasets gathered from online learning environments can address long-standing issues in chemical education research, such as the role of mathematics in the learning of chemistry explored here. There is strong evidence from past studies that students with lower preparation in mathematics have lower success rates in introductory college chemistry (Section 2). Here, analysis of more than 612 K student interactions with the Open Learning Initiative (OLI) General Chemistry courseware^1^ at a variety of two-year colleges allows us to decompose this link between mathematics and chemistry into two separate components: the initial knowledge of individual students on topics involving a high level of math, and the rate of learning on such topics. The overwhelming result is that it is only the initial knowledge that varies strongly across students and across the level of math involved in a particular topic. The student learning rate is nearly independent of both the level of math involved in a topic and the prior mathematical preparation of an individual student.

A significant feature of the current study is the use of large volumes of data gathered as students learn the content. This rich data has two potential advantages. First, data collected throughout the learning process provides more detail than studies that use pre- and post-tests to assess learning at only two time points. Second, data gathered through an entire semester enables analyses that examine student learning across a broad range of topics. Each practice opportunity in the courseware is labeled with one or more knowledge components (KCs) involved in the learning activity. KCs are an *“acquired unit of cognitive function or structure that can be inferred from performance on a set of related tasks”*.^2^ In this manner, the data may be viewed as including a pre- and post-test for each of the KCs in the first semester General Chemistry Course, along with data gathered in between.

A logistic regression is used to model student learning as a function of the number of interactions an individual student has had with a particular KC. We use the term “opportunities” — in the sense of “learning opportunities taken” rather than “learning opportunities provided” — to represent these interactions. This regression analysis generates estimates of both the initial knowledge and the learning rate for each student and each knowledge component. Here, the learning rate is defined in terms of the log of the odds that a student will complete a task without making an error or requesting a hint. The learning rate for a KC is the extent to which the log-odds increase each time a student engages with a task associated with that KC.

Another significant feature of this study is that the practice opportunities in the course are highly scaffolded through hints and feedback. Hints provide support upon request by the student, and feedback provides support when the student makes an error. The scaffolding fades as students move through a topic and picks up again with new topics. These scaffolds include support for quantitative reasoning, allowing the course materials to provide just-in-time support for mathematics. This just-in-time support likely has a strong influence on learning rates.

The current study builds on a recent finding from analysis of online practice data gathered within primary, secondary, and postsecondary mathematics, science, and language learning courses, which found that learning rates were “astonishingly similar” across students within these courses.^3^ Students’ initial knowledge varies widely, but their learning rate varies little. This study adds to these results by considering more nuanced questions related to the degree to which mathematics preparation influences the learning of chemistry content.

## 2 Literature Background

Mathematics is crucial in learning chemistry; it provides the tools needed to comprehend and manipulate the quantitative aspects of chemical reactions and phenomena. A large and growing body of research spanning nearly 100 years^4−6^ has established correlations between various measures of math preparation and success in general chemistry. In 1958, Kunhart, Olsen, and Gammons^7^ found high school chemistry and algebra grades to have higher correlations with course success (*R* = 0.26 and 0.20 respectively) than other more general measures of scholastic aptitude. In 1975, Pickering^8^ found a strong trend line between Math SAT scores and General Chemistry grades, and that a supplemental math/problem-solving course improved grades. In 1979, Ozsogomonyan and Loftus^9^ found that Math SAT scores had the highest correlation with course success (*R* = 0.51) followed by a chemistry pretest score (*R* = 0.42), high school chemistry grade (*R* = 0.38), and an algebra pretest score (*R* = 0.21). Predictions based on a combination of these factors showed strong correlation (*R* = 0.87) with course success.

Attempts to identify students who are less likely to successfully complete general chemistry continue to find that measures of math preparation, often along with measures of previous instruction in chemistry, are important factors in statistical models of course success.^10−23^ In 2021, Vyas et al.^24^ found that a combination of instruments could predict up to 80% of the students who did not pass the course.

Instruments aimed at measuring automaticity of arithmetical skills, such as the Math-Up Skills Test (MUST), have also found significant correlations with course success.^25−31^ For instance, Williamson et al.^27^ found that MUST scores correlated with course average (*R* = 0.54, *p* < 0.001) and that a logistic regression model that combined MUST with demographic variables could predict course success with 78% accuracy.

Such correlations prompted Sadler and Tai^32^ to suggest that ”two pillars” underpinning college STEM success are ”high school study in the same science subject and more advanced study of mathematics”. Below, we build on this by estimating students’ initial knowledge in both pillars: chemistry initial knowledge and math initial knowledge (Section 4.3).

One possible explanation for the observed correlations between chemistry course outcomes and prior math skills is that prior math knowledge aids the learning of chemistry by reducing the student’s cognitive load in processing chemistry instruction. Indeed empirical results and theory in cognitive science^33^ support the idea that, to accurately solve problems in the sciences, students should practice fundamental facts and procedures in a variety of mathematical and scientific contexts until they can be recalled and applied fluently and automatically.^34^ An alternative explanation is that prior math skill predicts course-relevant incoming knowledge and that incoming knowledge is, in turn, highly predictive of course outcomes. In other words, there is ambiguity in explaining this link from prior basic math knowledge to chemistry course outcomes as to whether or not prior basic math predicts learning (i.e., the difference between course-relevant incoming knowledge and outgoing knowledge) or predicts course inputs, which in turn predict outcomes because students with stronger incoming knowledge have less to learn.

The link between course preparation and course success also relates to policies regarding pre- and corequisite courses. Remedial courses have been widely recommended for students deemed to be underprepared (at-risk) in mathematics and/or chemistry, but evidence for their effectiveness is mixed. Some studies have reported increased success and retention with this approach, such as Donovan and Wheland^35^ and Stone et al.^21^ Others have found remediation efforts produced little to no short-term benefits, and sometimes had negative long-term consequences. For example, a six-year study by Gellene and Bentley^36^ concluded that a *“placement remediation program [was] providing little or no significant academic benefit,”* while further analysis by Jones and Gellene^37^ showed that this program increased attrition such that overall General Chemistry completion rates decreased. Attewell et al.^38^ found that taking remedial courses slightly decreased the likelihood that a four-year college student will complete a degree, but did not affect completion rates for two-year college students. A large-scale study by the U.S. Department of Education^39^ concluded that remedial courses could help or hinder, depending on students’ level of preparation, with only *“weakly prepared students who successfully completed all remedial courses”* benefiting overall. Shah et al.^40^ found that a parallel General Chemistry course with a corequisite support course improved outcomes for at-risk students in the first semester, but increased achievement gaps in the second semester. Denaro et al.^41^ reported improved performance on the final exam for students who took General Chemistry with a concurrent preparatory course. Sevian et al.^42^ found that coenrollment in a course that used an asset-based approach closed asymmetries when a nontraditional curriculum was used but not when a traditional curriculum was used. These mixed results provide further reason to better understand the correlations between math preparation and chemistry outcomes.

## 3 Methods

### 3.1 Data Collection

We present analyses of datasets collected in General Chemistry I courses at multiple sites across two semesters. We treat these two semesters as separate studies as a form of replication. In the Spring of 2021 (Study 1), data was collected from 183 students at four community colleges with four different instructors. In the following semester, Fall 2021 (Study 2), data collection included 279 students, ten instructors (11 classes), and seven community colleges. These hybrid courses were delivered in a classroom format with lectures and tests given by course instructors and interactive activities with hints and feedback implemented in the Open Learning Initiative (OLI) platform.^1^ The analyses presented below are based on the log data collected from the OLI portion of the course. The data and models used for analysis can be found in DataShop,^43^ a repository for educational data (datasets are ds4856 and ds5939 for Study 1 and 2, respectively). The experimental design and analyses were the same for both studies, though some content improvements were made to the Fall 2021 (Study 2) OLI course materials. This study was conducted with oversight by the Carnegie Mellon University Institutional Review Board (IRB), under protocol number STUDY2016_00000578.

Demographic information was collected from an optional survey provided within the courseware. In Study 1, about 55% of students responded, with 62% selecting an underrepresented (UR) race/ethnicity, 55% identifying as first-generation, and 61% selecting a gender other than male. In Study 2, about 48% of students responded, with 64% identifying as UR, 47% as first-generation, and 52% as a gender other than male. (Additional details are provided in the Supporting Information.)

The OLI General Chemistry courseware is based on the OpenStax textbook,^44^ and is intended to supplant textbook reading and online homework with a single integrated online platform. The content consists of modules that are roughly equivalent to a single textbook chapter. Modules include about 5 to 10 content pages, each of which includes didactic instruction, similar to the text and images in a textbook, “Learn by Doing” activities that typically break problem solving into steps and provide extensive hints and feedback to guide the student to the correct answer, and “Did I Get This?” activities that typically do not break the problem solving into steps and that provide less extensive scaffolding through hints and feedback. “Checkpoints” at the end of each module serve as homework quizzes, where feedback is given only at the end of the quiz. For all activity types, the measure of performance used in the logistic regressions is correct if students provided the correct response without asking for a hint, and incorrect otherwise. (Example activities are included in Supporting Information.)

### 3.2 Math Coding

Approximately 250 skills or knowledge components (KCs)^2^ were initially identified in the OLI General Chemistry 1 course. Each KC was categorized according to its math demand on a scale from 0 to 3 (see Examples of Math Coding in Supporting Information). Level 0 involves no math. Low math (level 1) typically involves a single calculation, such as converting between particles and moles. Medium math (level 2) generally involves several mathematical steps. For example, determining the empirical formula for a compound involves multiple gram-to-mole conversions, making a mole ratio (dividing all mole amounts by the smallest mole amount), then simplifying this to the smallest whole number ratio (which may involve converting a decimal value to a fraction). High math (level 3) usually involves algebra, including logarithms.

Math levels were coded independently by three experienced general chemistry instructors, and the average of their ratings was used as the math level for a given KC. When a coder gave a KC more than one rating, the average rating was used as their math level. For example, one coder rated the KC *Calculate the concentration of ions in solutions* as having both low (math level = 1) and medium (math level = 2) math demand, resulting in a combined math level = 1.5. The final math level code for this KC, in which the other two coders rated it as having a medium math demand (math level = 2), is the average of the 3 coders’ ratings ((2 + 2 + 1.5)/3 = 1.833).

Since many of the learning activities in the OLI General Chemistry 1 course involve multiple KCs, the KC model also contains about 100 concatenated KCs (i.e., combinations of 2 or more individual KCs). The math level of concatenated KCs is the largest math code of the individual KCs. For example, in a task labeled with the concatenated KC *Convert between mass and moles + Determine the empirical formula of a compound*, the former KC has a math level code = 1, and the latter KC has a math level code = 2; thus the concatenated KC is coded as math level code = 2 (i.e., medium math demand).

Inter-rater reliability (IRR)^45^ was assessed using a two-way mixed, consistency, average-measures intraclass correlation coefficient (ICC)^46^ to assess the degree to which coders provided consistency in their ratings of math level across knowledge components. The resulting ICC of 0.934 was in the excellent range,^47^ indicating that coders had a high degree of agreement, suggesting that math level was rated similarly across coders. The high ICC suggests that a minimal amount of measurement error was introduced by the independent coders, and therefore, statistical power for subsequent analyses is not substantially reduced. Math level ratings were therefore deemed to be suitable for use in the present study.

Approximately 10% of the knowledge components did not require any math (0 rating in Table 1), and more than three-fourths of the KCs involved low to medium math knowledge (i.e., 0.01–2 in Table 1). Relatedly, only 12% of the unique problem steps involve no math, and 6% have high math demand (i.e., greater than 2); thus, the remaining 82% involve a low to medium math demand (Table 1).

**Table 1:** Distribution of Math-Level Ratings for Study 1^a^

math rating	0	0.01–1	1.01–2	2.01–3
KCs (%)	33 (10%)	131 (38%)	132 (38%)	48 (14%)
steps (%)	454 (12%)	1627 (43%)	1494 (39%)	250 (6%)
opportunities per student KC	5.8	4.8	4.2	2.0
average performance	0.77	0.72	0.65	0.49
students	182	181	177	158

a Similar distributions were obtained for Study 2 (see the Supporting Information).

### 3.3 Research Design

To better understand how mathematics influences the learning of chemistry, we explore the following three research questions:

RQ1 Does lower prior preparation prevent or slow students in learning chemistry?
RQ2 Are Chemistry problems with high math content harder for students (a) to do or (b) to learn?
RQ3 Does lower prior math preparation prevent or slow students in learning chemistry?

To address these questions, we use variations on a logistic regression growth model^3^ that originated in the Additive Factors Model (AFM).^48^ AFM models are generalizations of Item Response Theory^49^ that add both a growth or learning element as well as a matrix encoding of a cognitive model^50^ of the underlying knowledge components (KCs) students need for successful task performance.

**Figure 1:**
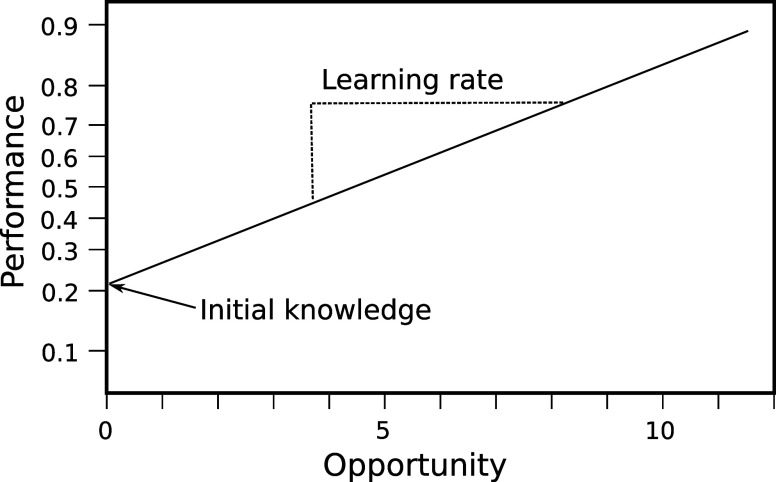
Schematic representation of the learning curves derived from regressions on student data. Predicted performance is on the *y*-axis, with labels that indicate the probability, *p*, that a student will succeed on a practice task without making an error or requesting a hint. The scale of the *y*-axis is log-odds, ln(*p*/(*p* – 1)), with *p* shown in the label. The *x*-axis is the number of practice opportunities to learn the KC the student has engaged with in the past.

The growth element included in the logistic regression can be visually represented as a learning curve, shown schematically in Figure 1. The *y*-axis is the probability that a student will succeed on a practice task, expressed as the log of the odds that the student will successfully complete the task without requesting a hint or making an error. The *x*-axis is opportunities, the number of times a student has interacted with tasks related to the associated knowledge component. For a student’s first interaction with a KC, the opportunity count is zero, to reflect past opportunities. Note that all students have access to the full courseware. We refer to the intercept of the regression line as the *initial knowledge*; it is similar to a pretest. We refer to the slope of the regression line as the *learning rate*, as it measures the increase in log odds of success for each opportunity taken. To aid interpretation of learning rate, we also convert this slope to % gain from 50%, i.e., the increase in performance that would be expected from this slope when performance is at 50%. Note that the regressions are performed on the population as a whole, which in effect, averages over various cohorts of students and various groupings of knowledge components.

As we move through the above research questions, we extend the logistic regression model of AFM to allow both initial knowledge and learning rate to depend on the level of math involved in a KC.

RQ1 investigates students’ initial knowledge and learning rate across all knowledge components to investigate if lower initial knowledge is associated with slower learning rates. This investigation is done using a modified AFM model called individual AFM or iAFM.^51^ The iAFM model includes a factor for individual student learning, δ_*i*_ of eq 1, that is not present in an AFM model. The logistic regression formula for iAFM is
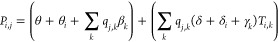
1where performance *P*_*i*,*j*_ is the log-odds, *ln*(*p*/(*p* – 1)), of the probability, *p*, that student *i* is correct on task *j*. The first term in parentheses models initial knowledge, and the second models learning rate. For initial knowledge, θ is the mean across all students and KCs, θ_*i*_ is the deviation from this mean for student *i*, and β_*k*_ is the deviation for the *k*th KC. The summation over KCs is included because each task can, in general, be associated with more than one KC through the matrix *q*_*j*,*k*_, which is 1 if task *j* involves KC *k* and 0 otherwise. However, here we instead treat tasks involving multiple knowledge components by labeling them with a new KC that concatenates the individual KCs (Section 3.2). The learning rate is the slope of performance with respect to *T*_*i*,*k*_, where *T*_*i*,*k*_ is the number of past opportunities student *i* has had to interact with a task involving KC *k*. The learning rate is modeled with an overall mean, δ, and deviations for an individual student, δ_*i*_, and KC, γ_*k*_.

For research question 2 (RQ2), we explore the impact of math difficulty on initial knowledge and learning rate. Here, we add math level (based on the coding of KCs described above) as an independent variable to the iAFM model to estimate the dependence of initial knowledge and learning rate on math level,
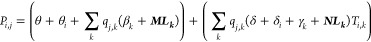
2where *L*_*k*_ is a continuous variable, with a range of 0 to 3, describing the math level of the *k*^*th*^ KC (Section 3.2), while *M* and *N* describe, respectively, the dependence of initial knowledge and learning rate on *L*_*k*_.

In research question 3 (RQ3), we examine the effects of prior math preparation on learning chemistry. To do so, the dependence of initial knowledge on math level is allowed to vary by student,
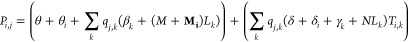
3where *M*_*i*_ describes the degree to which the initial knowledge of student *i* depends on math level, *L*_*k*_. We take *M*_*i*_ as a measure of an individual student’s math preparation because larger magnitudes indicate that the *i*th student’s initial knowledge on a KC depends strongly on the level of math associated with that KC. We refer to θ_*i*_ from eq 3 as an individual student’s chemistry preparation, as it measures the *i*^*th*^ student’s initial knowledge extrapolated back to math level *L*_*k*_ = 0.

An additional investigation for RQ3 involves math preparation and learning opportunities: Do students with higher math preparation take greater advantage of learning opportunities by doing more problem steps? Or, conversely, do students with lower math preparation, who need more practice, do fewer problem steps?

The regressions are performed using the generalized linear mixed-effect model (glmer) function in the R Statistical Software.^52^ The regression formulas and other details are in the Supporting Information.

## 4 Results

We applied regression analyses as indicated by eqs 1–3 to the two datasets of Section 3.1. For each research question, we first discuss the detailed results from the Study 1 dataset. We then assess the extent to which the analysis of the Study 2 dataset aligns with these findings.

### 4.1 RQ1: Does Lower Prior Preparation Prevent or Slow Students in Learning Chemistry?

**Figure 2:**
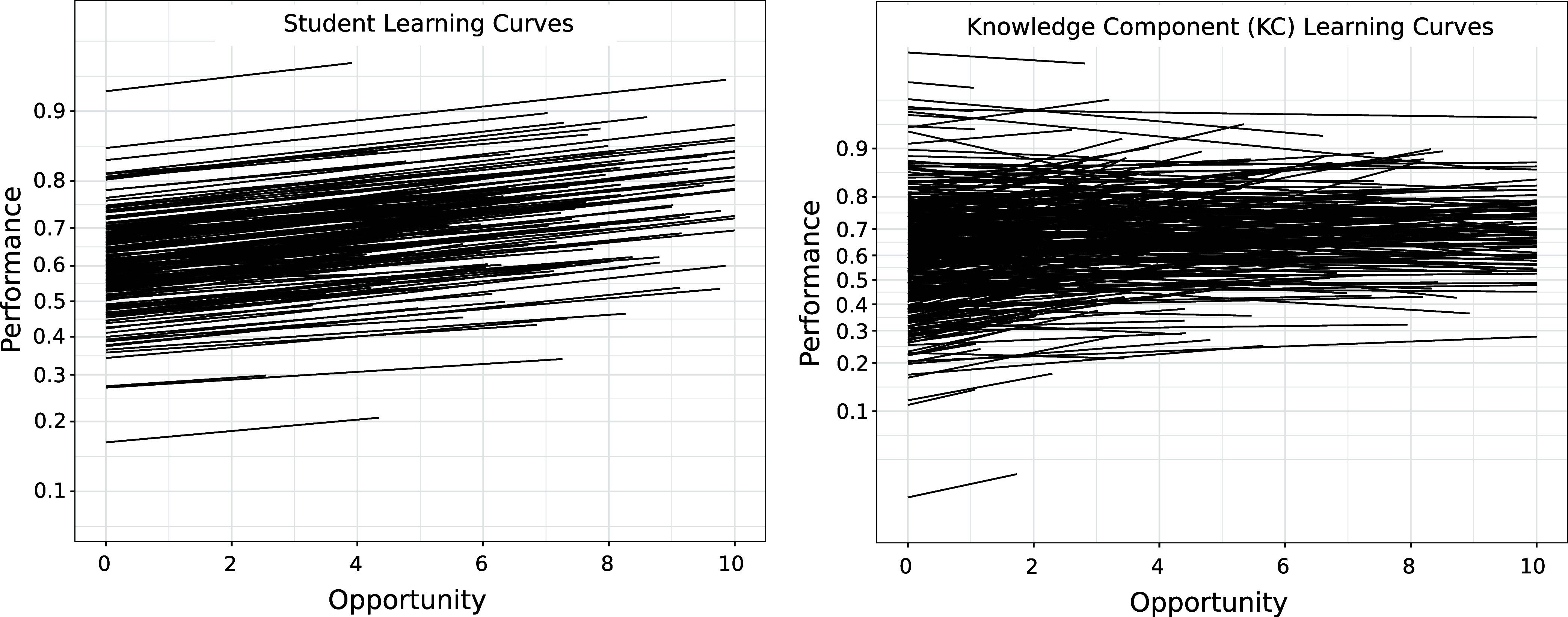
Student learning curves (left panel) show a large variation in initial knowledge, as indicated by the wide range of intercepts, but small differences in learning rate, as indicated by the mostly parallel slopes. The range of intercepts on the KC learning curves (right panel) shows a large variance in initial knowledge, and the slope variance suggests some KCs are learned faster than others. The axes are as in Figure 1.

To address RQ1, we use learning curves to visualize the parameter estimates resulting from applying the logistic regression of eq 1 to the dataset for Study 1. The regression parameters can be used to generate a learning curve for each student, with θ + θ_*i*_ for initial knowledge and δ + δ_*i*_ for learning rate (left panel of Figure 2). The large spread of intercepts on the *y*-axis indicates wide variation in students’ initial knowledge (interquartile range, IQR = 54%, 69%). In contrast, the nearly parallel lines seen in the learning curves indicate only small differences in student learning rates. Thus, the less prepared students (lines that start lower on the *y*-axis) are learning chemistry at a rate similar to that of the better prepared students (lines that start higher on the *y*-axis have about the same slope).

The right panel of Figure 2 shows learning curves for each knowledge component in the course. These curves are generated from the parameter estimates from eq 1 using θ + β_*k*_ for initial knowledge and δ + γ_*k*_ for learning rate. Here, the regression essentially averages over all students to generate a learning curve for each knowledge component. As seen above for students, there is a large variation in the difficulty of knowledge components as indicated by the wide range of initial knowledge (IQR = 48%, 70%). However, unlike students, there is also substantial variation in learning rates, as indicated by the nonparallel lines in the right panel of Figure 2. Some initially hard KCs are learned more quickly and others are learned more slowly. For example, the KC *Predict differences in melting points of ionic substances* has an initial starting point (i.e., intercept) of 34%, thus quite difficult. It improves at 0.11 log odds (about 2.6% from 50%) per opportunity. However, the (also) hard KC *Calculate changes in vapor pressure based on concentration of solution*, with an intercept of 37%, only improves 0.036 log odds (about 0.90% from 50%) per opportunity. In other words, both KCs are hard, but *Predict differences in melting points of ionic substances* is learned more quickly than *Calculate changes in vapor pressure based on concentration of solution*. Similarly, some initially easier KCs are learned more quickly and others are learned more slowly.

Similar results are found when this analysis is applied to the dataset for Study 2 (see Supporting Information). The learning curves for individual students show large variation in initial knowledge (IQR = 54%, 71%) with nearly parallel lines indicating small variations in learning rates. The learning curves for individual KCs shows large variations in both initial knowledge and learning rates.

We take students’ initial knowledge as a measure of their prior preparation. Both studies find that all students tend to learn at nearly the same rate, suggesting that prior preparation does not hinder or slow students’ ability to learn chemistry. However, there are significant differences in the rates at which different KCs are learned. In RQ3, we further decompose initial knowledge into separate measures of chemistry and math preparation to explore their individual contributions.

### 4.2 RQ2: Are Chemistry Problems with High Math Content Harder for Students (a) to do or (b) to Learn?

Having established that initial knowledge varies significantly among students while learning rates remain largely consistent, we now turn our attention to examining how the mathematical complexity of a knowledge component (KC) impacts both initial knowledge and learning rate. To address this, we extend the logistic regression to that of eq 2, which adds a dependence on the level of math involved in a particular KC when estimating both initial knowledge (*M* of eq 2) and learning rate (*N* of eq 2). The results in Table 2 for the Study 1 dataset indicate a statistically significant dependence of initial knowledge on math level (*M*; *p* < 0.001). The dependence of learning rate on math level is not significant (*N*; *p* = 0.083), but it is notable that the trend is for content with a high math level to be learned faster than content with a low math level.

**Table 2:** Results of the Impact of Math on Initial Knowledge and Learning Rate from the Regression Analysis of eq 2

	**estimate (std error)**	***p* value**
**Study 1**		
initial knowledge (θ)	1.172 (0.11)	<2 × 10^–16^
learning rate (δ)	0.041 (0.02)	0.011
initial knowledge by math level (M)	–0.582 (0.07)	<2 × 10^–16^
learning rate by math level (N)	0.021 (0.01)	0.083

**Study 2**		
initial knowledge (θ)	1.198 (0.10)	<2 × 10^–16^
learning rate (δ)	0.081 (0.02)	6.39 × 10^–5^
initial knowledge by math level (M)	–0.574 (0.07)	3.61 × 10^–16^
learning rate by math level (N)	0.011 (0.01)	0.446

Table 3 uses the regression parameters of Table 2 to summarize the impact of math level (column 1) on initial knowledge (column 2) and learning rate (columns 3 and 4). Initial knowledge has a strong and statistically significant dependence on math level, with initial knowledge for the typical student being about 76% correct for chemistry KCs with no math (math level = 0), dropping to 36% correct for KCs with the highest math level (math level = 3). The overall learning rate of δ = 0.041 log odds gain per opportunity is statistically significant (*p* = 0.011). Table 3 also reflects a dependence of learning rate on math level, but this dependence is not statistically significant (*p* = 0.083).

**Table 3:** Impact of the Math Level of Knowledge Components on Student Initial Knowledge and Learning Rate^a^.

math level	initial % correct	log odds gain per opportunity	% gain from 50%^b^
**Study 1**			
0	76%	0.041	1.0%
1	64%	0.062	1.6%
2	50%	0.084	2.1%
3	36%	0.105	2.6%

**Study 2**			
0	77%	0.081	2.1%
1	65%	0.092	2.2%
2	51%	0.104	2.6%
3	37%	0.115	2.9%

a Note that the statistical significance of the increase in learning rate with the math level is marginal (*p* = 0.083 in Study 1 and 0.446 in Study 2).

b We convert log odds gain to percent correct from 50% (e.g., 0.041 to 51.0% in row one) and then subtract 50%.

**Figure 3:**
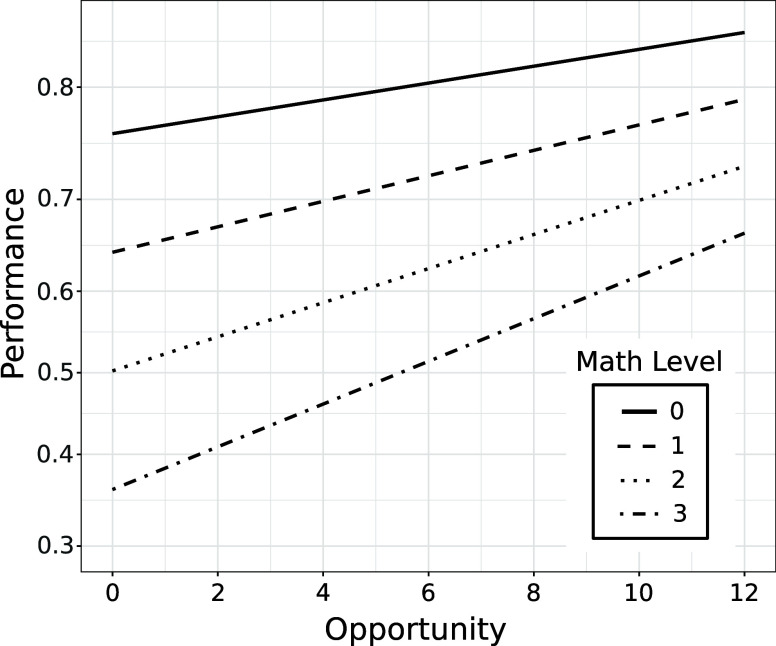
Learning curves generated from the regression parameters for study 1 in Table 3. Initial knowledge decreases dramatically with math level. The learning curves are steeper for higher math level but this increase in learning rate is only marginally statistically significant (see Table 3). The results do however indicate that students are learning high math content at least as readily as they are learning low math content. The axes are as in Figure 1.

Figure 3 provides a complementary visualization by using the regression parameters to generate learning curves for math levels 0 through 3. As illustrated, performance on KCs with higher math levels starts off lower but students are learning chemistry skills and concepts involving harder mathematics (levels 2 and 3) as readily as they are learning skills and concepts involving little or no math.

The lower sections of Tables 2 and 3 present the results from applying this analysis to the Study 2 dataset. Consistent with Study 1, initial knowledge shows a strong and statistically significant dependence on math level (*M*; *p* < 0.001). Also consistent with Study 1, there is no statistically significant dependence of learning rate on math level (*N*; *p* = 0.446). In both studies, we found no evidence that the increased math level of a KC slows the learning rate when averaged across all students.

Although neither study finds a significant dependence of learning rate on math level, there is a statistically significant difference in learning rate between Study 1 and Study 2. When the data from the two studies is combined and the study-ID is added as an independent variable to the regression analysis for RQ2, the only significant interaction we find is that the learning rate for Study 2 is 17% larger than for Study 1 (*p* = 1.3 × 10^–8^, see Supporting Information). This can be rationalized in terms of Study 2 being conducted on courses offered in the fall semester while Study 1 is for courses offered in the spring. Students in Study 2 are therefore taking the course on-sequence and tend to be better prepared, often having taken a prior chemistry course that covers some of the content in a college general chemistry course. It is interesting that the differences are significant only for learning rate and not initial knowledge. This is consistent with past exposure to the topics in the course having a stronger impact on the facility with which students can relearn that material than their ability to recall it.^53^ A similar rationale—that previous exposure to the content of this course can enhance learning rate—will be invoked in Section 4.3 below, to account for an association between learning rate and initial chemistry knowledge.

Next, we explore how individual differences in math preparation might influence student learning rates.

### 4.3 RQ3: Does Lower Prior Math Preparation Prevent or Slow Students in Learning Chemistry?

**Figure 4:**
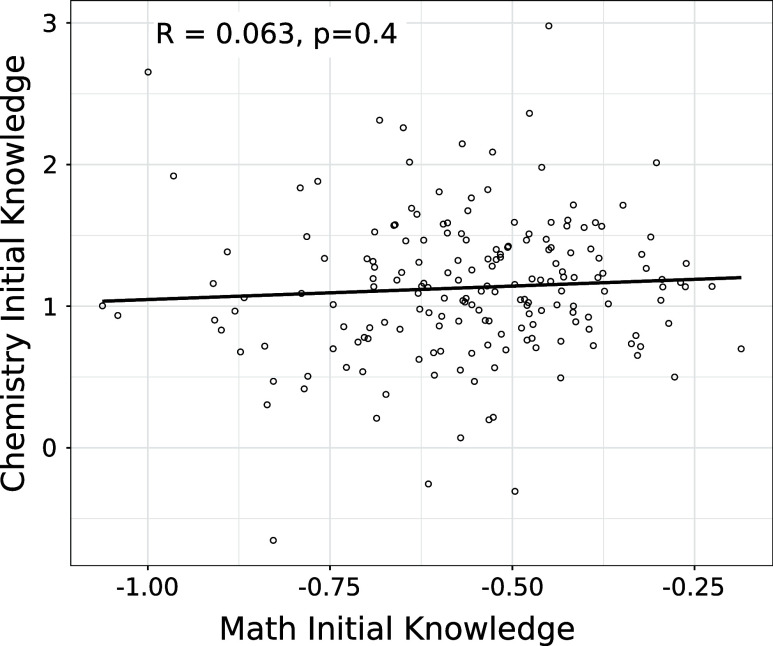
Study 1 estimates of students’ Math Initial Knowledge (*M* + *M*_*i*_ of eq 3) and Chemistry Initial Knowledge (θ + θ_*i*_ of eq 3) are quite independent of each other (*R* = 0.06) in contrast to the expectation that students with more math preparation are likely to have more chemistry preparation.

In addressing RQ2, the logistic regression included a dependence of initial knowledge on the level of math involved in a particular KC through the term *M* of eq 2. This *M* was an average over all students. To address RQ3, we extend this to include a dependence on individual students via the term *M*_*i*_ of eq 3. This additional factor allows us to extrapolate initial knowledge back to math level zero, *L*_*k*_ = 0 in eq 3, obtaining an estimate, θ + θ_*i*_, for initial knowledge of the *i*th student on knowledge components that involve no math. We refer to this extrapolated value, θ + θ_*i*_, as the *chemistry initial knowledge* of that student. The dependence of the *i*th student’s initial knowledge on math level is estimated in eq 3 as *M* + *M*_*i*_, which we refer to as *math initial knowledge*. Chemistry and math initial knowledge are strikingly uncorrelated, *R* = 0.063 for the Study 1 dataset (Figure 4). Next, we separately examine correlations of math and chemistry initial knowledge with learning rates and other factors.

In addressing RQ2, the logistic regression included a dependence of initial knowledge on the level of math involved in a particular KC through the term *M* of eq 2. This additional factor enabled us to examine how students’ initial knowledge and learning rate varies with the mathematical demands of different KCs. Because *M* was an average over all students, the finding that initial knowledge has a significant dependence on math level is for the population as a whole. To address RQ3, we extend this to individual students via the term *M*_*i*_ of eq 3. This allows us to separate the dependence of initial knowledge on math into *M*, the average for the population as a whole, and *M*_*i*_, the deviation from this average for each individual student. We refer to *M* + *M*_*i*_ as the *math initial knowledge* of an individual student. Because, in the regression of eq 3, the dependence of initial knowledge on math level has been accounted for in *M* + *M*_*i*_ of eq 3, we refer to θ + θ_*i*_ of eq 3 as the *chemistry initial knowledge* of the individual student.

Chemistry and math initial knowledge are strikingly uncorrelated, *R* = 0.063 for the Study 1 dataset (Figure 4). Next, we separately examine correlations of math and chemistry initial knowledge with learning rates and other factors.

**Figure 5:**
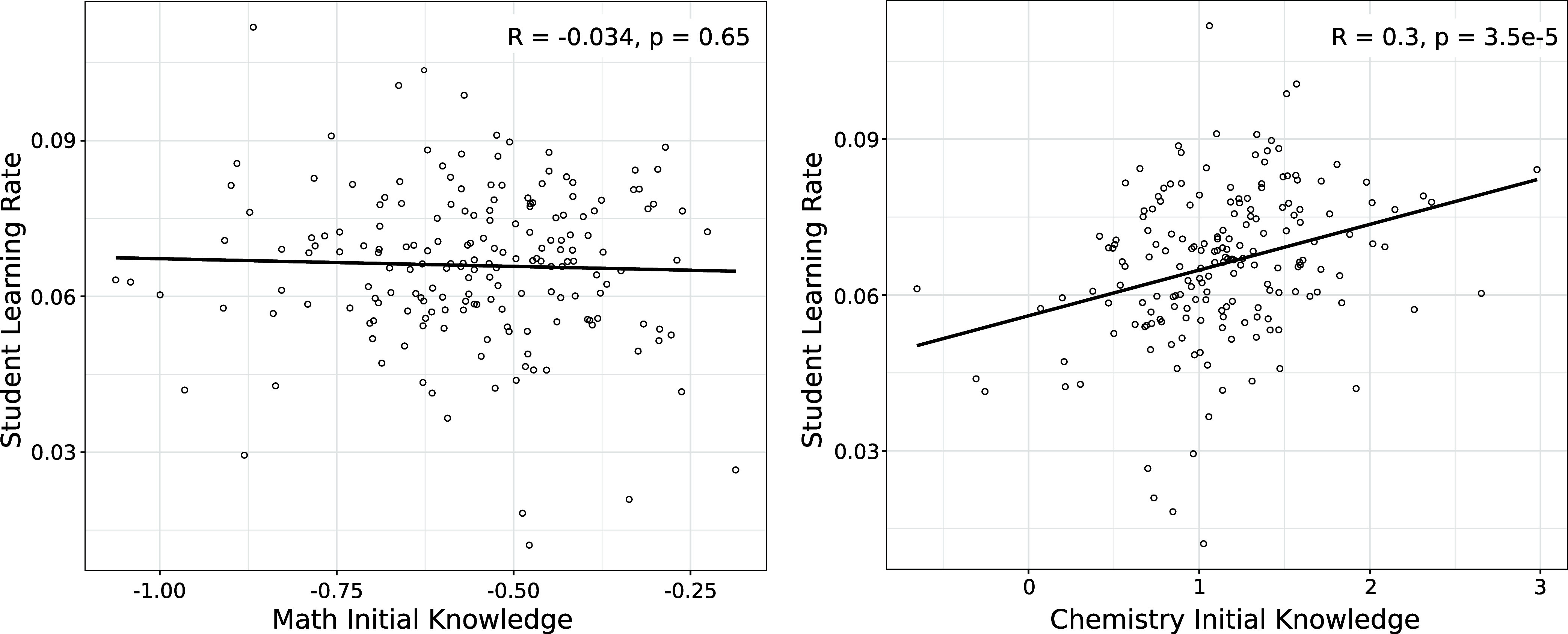
Left scatterplot shows that math initial knowledge is not associated with student learning rate (flat slope), while the right scatterplot indicates that chemistry initial knowledge is associated with student learning rate (upward slope).

To determine whether lower math preparation prevents or slows student learning in chemistry, we computed the correlation between individual student learning rate, δ + δ_*i*_ of eq 3, and math initial knowledge, *M* + *M*_*i*_ of eq 3. We found no correlation, *R* = −0.034, *p* = 0.65 (Figure 5 left panel). In other words, students with lower prior math preparation learn chemistry at a rate equivalent to students with higher math preparation.

We also investigated whether chemistry initial knowledge is associated with individual student learning rates, δ + δ_*i*_ of eq 3. Here, we found a statistically significant positive correlation, *R* = 0.30, *p* < 0.0001 (Figure 5 right panel). Students with lower chemistry initial knowledge (below median) do learn at a somewhat slower rate (slope = 0.062 log odds or about 1.5% gain per opportunity) than students with higher incoming chemistry knowledge (above median: slope = 0.070 log odds or about 1.9% gain per opportunity). This significant correlation helps confirm that individual learning rates are picking up true student-level variability, not random noise. Thus, it lends confidence to the inference that a lack of prior math knowledge does not slow chemistry learning (i.e., the lack of correlation between math initial knowledge and learning rate cannot be attributed to a lack of variability in learning rate). The faster learning rate observed in students with higher initial chemistry knowledge may be attributed to their familiarity with closely related content from previous chemistry courses, allowing them to relearn the material, whereas students with lower initial knowledge may be encountering this content for the first time.

We give a sense of the practical significance of the correlation between chemistry initial knowledge and learning rate by inspecting differences between the typical less prepared student (i.e., the first quartile) and the typical more prepared student (i.e., the third quartile). A typical less prepared student is estimated to start at about 57% correct on math level 1 tasks and has a learning rate (based on the correlation in Figure 5) of 0.063 log odds (about 1.5% gain from 50%) per opportunity. At this learning rate, they need about 17 opportunities to reach a reasonable mastery level of 80%. If these students had the slightly higher learning rate of the typical more prepared student, namely, 0.068 log odds (about 1.7% gain from 50%), they would need about 16 opportunities, that is, only about one less opportunity. Thus, the challenge for less prepared students is not the rate at which they are learning but the fact that they have more to learn.

We next examine the relation between initial knowledge and the number of opportunities taken. Recall that the opportunity variable refers to the number of interactive learning opportunities students engage in, not the total available (which is the same for all students). There is a small and significant correlation between math initial knowledge and learning opportunities (*R* = 0.21, *p* = 0.005). A similar small and significant correlation exists between chemistry initial knowledge and opportunities (*R* = 0.24, *p* = 0.001). This effect of initial knowledge on opportunities is larger than the effect of initial knowledge on learning rate. Similar to the above, we inspect differences in opportunities taken between the typical less prepared student (i.e., the first quartile) and the typical more prepared student (i.e., the third quartile). A student at the first quartile in math initial knowledge (*M* + *M*_*i*_ = −0.65 log odds; 34%) engages in about 1300 practice opportunities, whereas a student at the third quartile (−0.44 log odds; 39%) in math initial knowledge engages in about 1570 opportunities. In other words, the top half of students in math initial knowledge do about 270 (21%) more opportunities than the bottom half of students in math initial knowledge.

Likewise, a student at the first quartile in chemistry initial knowledge (0.84 log odds; 69.9% for level 0 math content) is estimated to take 1280 opportunities, whereas a student at the third quartile (1.41 log odds; 80.4% for level 0 math content) in chemistry initial knowledge does about 1560 opportunities. Thus, the top half of students in chemistry initial knowledge complete about 280 (22%) more opportunities than the bottom half of students in chemistry initial knowledge.

In a follow-up regression analysis (see Supporting Information) we found that students’ chemistry and math initial knowledge both have a significant and independent association with students’ total practice opportunities. This result is consistent with the idea that students coming into the course less well-prepared in either mathematics or general chemistry end up pursuing fewer opportunities. This result is perplexing given the high similarity in learning rates: *Lower initial knowledge students pursue fewer opportunities even though they are making progress at about the same rate as students with higher prior chemistry and math preparation.*

Similar results are obtained when the analyses for RQ3 are applied to the Study 2 dataset. As in Study 1, chemistry initial knowledge and math initial knowledge show only a weak and insignificant correlation (*R* = 0.079, *p* = 0.19). Also as in Study 1, we find math initial knowledge is not associated with learning rate (*R* = 0.045, *p* = 0.46), but chemistry initial knowledge is positively correlated with learning rate (*R* = 0.40, *p* = 4.2 × 10^–12^). The difference in learning rate for students in the lower half of chemistry initial knowledge versus their counterparts in the upper half are 0.089 versus 0.099 log odds gain per opportunity. These learning rates in more intuitive terms are 2.2% gain versus 2.5% gain from 50%, respectively.

Also as in Study 1, Study 2 finds a small and significant correlation between math initial knowledge and the number of learning opportunities students chose to engage with (*R* = 0.171, *p* = 0.004). We make these relationships more concrete as follows. A typical student at the first quartile in prior math (*M + M*_*i*_, which is −0.66 log odds; 34.1%) is estimated to engage in about 1180 opportunities whereas a student at the third quartile in prior math (−0.47 log odds; 39.6%) engages in about 1401 opportunities. In other words, the top half of students in math preparation do 221 (18.7%) more opportunities than the bottom half of students in math preparation.

In Study 1, the correlation between chemistry initial knowledge and opportunities taken was similar to that of math initial knowledge. In Study 2, we instead find that the correlation between chemistry initial knowledge and opportunities taken is not significant (*R* = 0.003, *p* = 0.97). A typical student at the first quartile in prior chemistry (*θ + θ*_*i*_ which is 0.86 log odds; 70.4% for level 0 math content) takes an estimated 1276 opportunities whereas a typical student at the third quartile (1.52 log odds; 82.0% for level 0 math content) takes an estimated 1280 opportunities. Consistent with the lack of correlation, there is no meaningful difference (at 4 opportunities and 0.3%) in opportunities taken.

From both studies, we find the challenge for less prepared students is not that they have trouble learning chemistry–they are learning at essentially the same rate as better prepared students. The challenge is that they need more learning opportunities to reach a given performance level. Both studies also find a small but statistically significant correlation between math initial knowledge and number of opportunities taken, while only Study 1 finds a significant correlation of chemistry initial knowledge with opportunities taken.

## 5 Discussion

Unquestionably, student prior math preparation has a substantial influence on their final success in introductory college chemistry courses. Here, we use data gathered as students engage with online learning materials to first examine initial knowledge and learning rates for both students and knowledge components (RQ1) and then decompose this link between preparation and course success into five components: initial knowledge in math and chemistry, learning rate for math and chemistry, and learning opportunities taken. We first summarize our findings and then consider possible implications for course and curriculum design.

### 5.1 Summary

Our results suggest that the challenge for less prepared students is not the rate at which they are learning, but rather the fact that they come in with less initial knowledge (Section 4.1). The analysis shows that initial knowledge, averaged over the various knowledge components (KCs) of the course, varies substantially among students. The learning rates are, however, surprisingly similar across students. These results are consistent with findings in 27 other datasets of student learning in course-embedded use of online practice in science, math, and language courses where student learning rates were found to be surprisingly similar.^3^ As we observed here, this similarity in learning rate is particularly striking in comparison to the high variability in student initial knowledge.

The similarity in learning rate across students is also striking in comparison to the high variability in learning rate across knowledge components (KCs). Because student learning rate is relatively constant, the difference in learning rates among KCs is not a result of better prepared students learning harder KCs more quickly and less prepared students learning them more slowly. The differences are likely due to either: differences in the complexity of the KC that impact its acquisition (e.g., the Clausius–Clapeyron equation is a more complex formula than Boyle’s law) or imperfections of the labeling of tasks with KCs (e.g., some of the tasks labeled with the same KC may have other knowledge requirements that have not been considered and when these tasks are in later opportunities they flatten the learning curve). In either case, identifying KCs with slow learning provides a direct means to guide iterative improvement in the learning resources.^51,54^

We also investigated whether Chemistry problems with high math content are harder for students to do or to learn (RQ2). Our results suggest the level of challenge for students increases substantially with the level of math involved in a particular KC, which is consistent with past studies showing a link between math preparation and course success. However, we again find that this challenge is related to initial knowledge not learning rate. Initial success is estimated at about 75% accuracy on no-math tasks and at about 36% on the highest math level tasks. Surprisingly, higher math level does not seem to inhibit learning rate, there is no statistically significant dependence of learning rate on math level in either study.

Finally, we explored whether lower prior math or chemistry preparation prevents or slows student learning (RQ3). Our results suggest that students’ math initial knowledge also has little influence on their learning rate. This investigation was done by decomposing initial knowledge into math initial knowledge, as measured by the dependence of a students’ initial knowledge on the level of math involved in a particular KC, and chemistry initial knowledge, as measured by estimating initial knowledge on KCs with math difficulty factored out. It is interesting that these two components of initial knowledge show little correlation with one another. It is also interesting that student learning rate shows a statistically significant correlation with chemistry initial knowledge but not math initial knowledge. This positive correlation of learning rate with initial chemistry knowledge is, however, quite small, with initial knowledge remaining the primary source of challenge for less well-prepared students.

Small correlations were found between the number of opportunities taken and initial math knowledge (*p* = 0.005 for Study 1 and *p* = 0.004 for Study 2) and the number of opportunities and chemistry initial knowledge (only Study 1 was significant with *p* = 0.001).

Even though less prepared students make progress at almost the same rate, they tend to engage with somewhat fewer learning opportunities. Although students with lower initial knowledge *can and do succeed*, some *may believe they cannot* and therefore participate less.

### 5.2 Limitations

This study has several limitations that should be considered when interpreting the results. First, the data analyzed in this study were gathered exclusively from interactions within the courseware. As a result, key instructional activities such as in-class lectures, instructor office hours, and peer discussions were not captured. Consequently, our findings do not account for the potential impact of these additional learning experiences on student performance.

Furthermore, the study does not include performance data from assessments conducted outside the courseware, such as exams that play a significant role in determining students’ final grades. The exclusion of these assessments limits our ability to evaluate how engagement with the courseware translates to broader course success.

Another important limitation is the lack of data on students who opted out of the study. We do not have information on the number of students in each class who chose not to participate, nor do we have insights into their demographic or academic characteristics. This introduces the possibility of selection bias, which may influence the generalizability of our findings.

Finally, we do not have data on external factors that could affect student engagement with the courseware, such as access to computing resources. Limited or inconsistent access to necessary technology may have impacted the number of opportunities students took within the courseware and, by extension, their learning outcomes.

Despite these limitations, the findings provide valuable insights into student learning patterns and the role of prior math preparation in chemistry education. Future work could explore these external factors to provide a more comprehensive understanding of student success.

### 5.3 Implications

The observation that the student challenge lies primarily in initial knowledge, as opposed to learning rate, may have implications for course and curriculum design. This aligns with research on growth mindset, which emphasizes that abilities and competence develop through effort and persistence.^55^ In this context, providing students with additional opportunities to engage with chemistry concepts may foster a learning environment that encourages resilience and long-term success. Additionally, making students aware of their learning rates—by explicitly sharing progress data—may help reinforce the idea that improvement is occurring, even if mastery takes time.

These findings also have implications for how best to support students with weaker math backgrounds. If low math preparation slowed learning, then it would seem advisible to have less well-prepared students take a math preparation course before enrolling in chemistry. However, if, as observed here, low math preparation does not slow learning, then providing math support before entering chemistry may not be necessary. Rather, providing support through math-specific instructional feedback and hints on tasks involving math, as is done in the courseware employed in this study, appears a highly viable alternative.

Although no link is observed here between initial knowledge and learning rate, this does not imply that students with lower preparation do not need additional support to achieve course success. Even if learning rates are the same, less well-prepared students will require substantially more opportunities, and thus time, to succeed. For example, the left panel of Figure 2 shows that, averaged over all KCs, students who start with low initial performance remain below the *initial* performance of many of their better prepared peers even after engagement with the courseware. From Table 3, we estimate an average learning rate of approximately 0.07 log-odds per opportunity. Assuming this learning rate, students whose initial performance is at the lower and upper bounds of the interquartile range (IQR of 54 and 69%) will achieve performances of 70 and 82%, respectively, after 10 opportunities. Notably, 10 opportunities exceed the average number of opportunities students take per KC. However, this is only sufficient to move a student who starts at the lower bound of the IQR to the initial performance of a student who starts at the upper bound. This suggests that students require substantially more time than is currently available to achieve high performance.

A systemic approach to providing this additional time is not consistent with most current curricula, which assign credit hours based on content and without consideration of student needs. The need for educational systems that include support for this extra learning time is further supported by the results reported here: Students with less preparation tend to take somewhat fewer opportunities to engage with the material. The reasons for this remain to be investigated. We suggest exploration of three candidate hypotheses. First, individual opportunities may take less well-prepared students longer to complete, because the scaffolding provided in the opportunities takes time to engage with. Second, the life circumstances that lead some students to enter the course with lower initial knowledge may also lead to less available time to engage with learning materials during the course. Third, some students may find it discouraging to experience errors, even if they are learning from them^56^ and thus decide to disengage. This third reason suggests putting more attention to supporting student motivation and perhaps more explicit statements that every learner makes errors and it is a natural part of the learning process.

In general, we advocate efforts to free up time for students by making their learning more efficient. The observation that learning rates are similar across all students does not imply that improvements in instruction cannot speed learning. Learning rate does vary substantially across KCs (Figure 2), with many KCs showing slow learning. We recommend applying methods for identifying KCs with slow learning and modifying the relevant instruction (e.g., by tailoring new practice tasks and instruction that directly address cognitive challenges) to improve learning efficiency and effectiveness.^51,54^

More generally, this effort illustrates the potential for analysis of fine-grained longitudinal data, gathered in authentic educational settings, to address century-old research questions in the learning of chemistry. A large and expanding collection of such data is available through LearnLab’s DataShop along with tools to support analysis.^43^

## Supporting Information Available

Example instructional activities and associated math coding; equations in R used for the logistic regressions; regression analyses of math and chemistry initial knowledge effects on opportunities (Section 4.3); and additional results from study 2 (PDF)
