# Supplementary material for: Mathematics Matters or Maybe Not: An Astonishing Independence between Mathematics and the Rate of Learning in General Chemistry
Source: JACS Au. 2025 Feb 26;5(3):1268–78. doi: 10.1021/jacsau.4c01126 (PMC11938002; doi:10.1021/jacsau.4c01126)
Supplement: Supplementary file 1 — au4c01126_si_001.pdf [file au4c01126_si_001.pdf]

Supporting Information: Mathematics Matters or Maybe Not: An Astonishing Independence Between Mathematics and Rate of Learning in General Chemistry

Kenneth R. 2010,[†] Mark Blaser,[‡] Elizabeth A. McLaughlin,[†] Hui Cheng,[†] and David J. Yaron[*,¶]

†*Human Computer Interaction Institute, Carnegie Mellon University, Pittsburgh, PA*

‡*The Simon Initiative, Carnegie Mellon University, Pittsburgh, PA*

¶*Department of Chemistry, Carnegie Mellon University, Pittsburgh, PA*

E-mail: yaron@cmu.edu

S1 Example activities with Math Coding

As discussed in the main manuscript, the math levels for each knowledge component (KC) were determined by averaging scores assigned by three reviewers, resulting in an intraclass correlation coefficient (ICC) of 0.934. Below, we present examples of learning activities associated with KCs that span a range of math demands. It is important to note that the math code is assigned to the KC as a whole, rather than to individual activities.

The examples provided include both "Learn By Doing" and "Did I Get This?" exercises. "Learn By Doing" activities typically appear earlier in the instructional sequence and offer more extensive hints and feedback, while "Did I Get This?" exercises come later and provide less scaffolding. In both cases, the performance measure used in the logistic regressions is binary: a response is marked correct if the student answers correctly without requesting a hint, and incorrect otherwise.

The illustrations below demonstrate the types of scaffolding provided during the activities. Blue boxes represent hints, pink boxes display feedback after an incorrect response, and green boxes show feedback after a correct response.

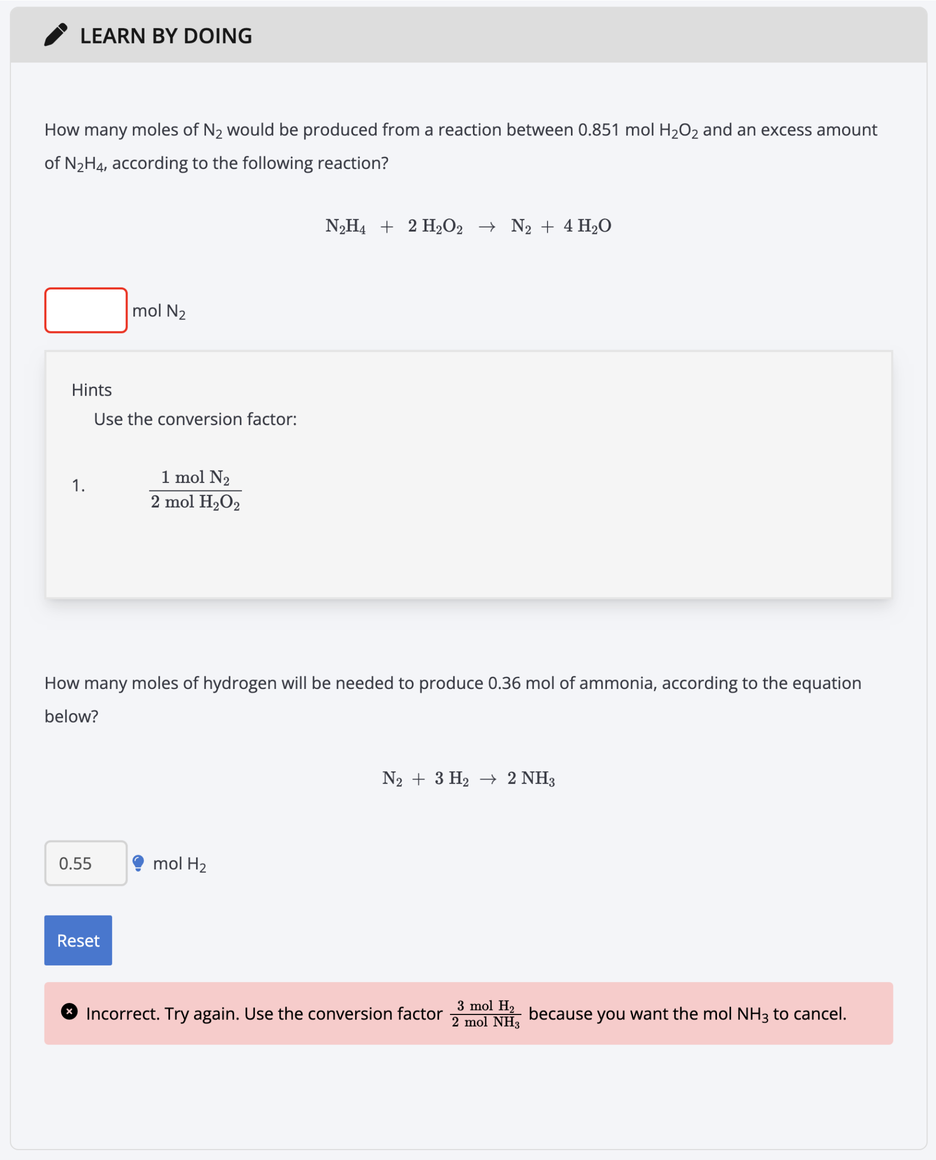

S1.1 Math level 0 (no math)

No math is involved. Numbers may be mentioned, such as boiling temperature, but no calculations are performed.

KC: *Distinguish among pure substances (math level = 0)*

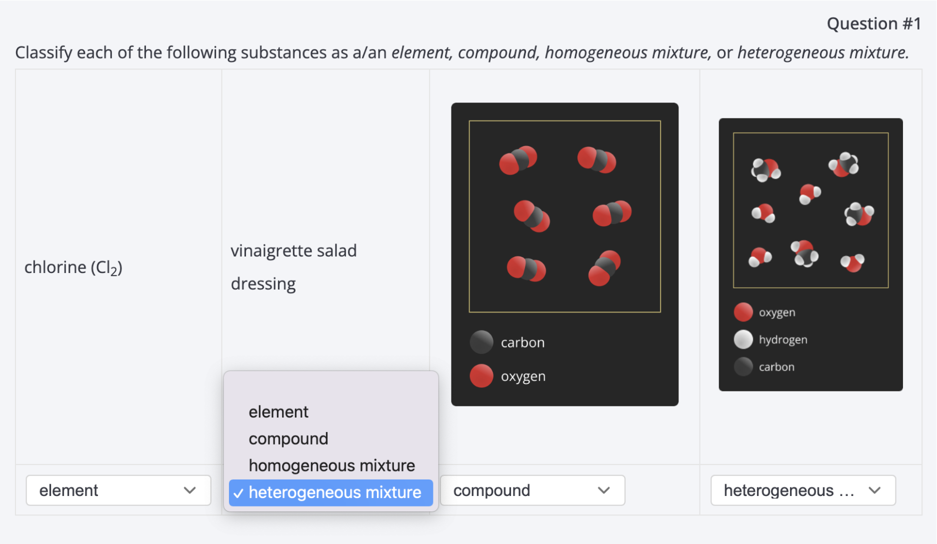

S1.2 Math level 1 (low math)

Low math skills generally involve a single calculation. For example, converting between particles and moles is often a one-step process that requires setting up a conversion factor and performing a multiplication or division. Alternatively, this conversion can be completed using simple proportions.

KC: *Convert between particles and moles (math level = 1)*

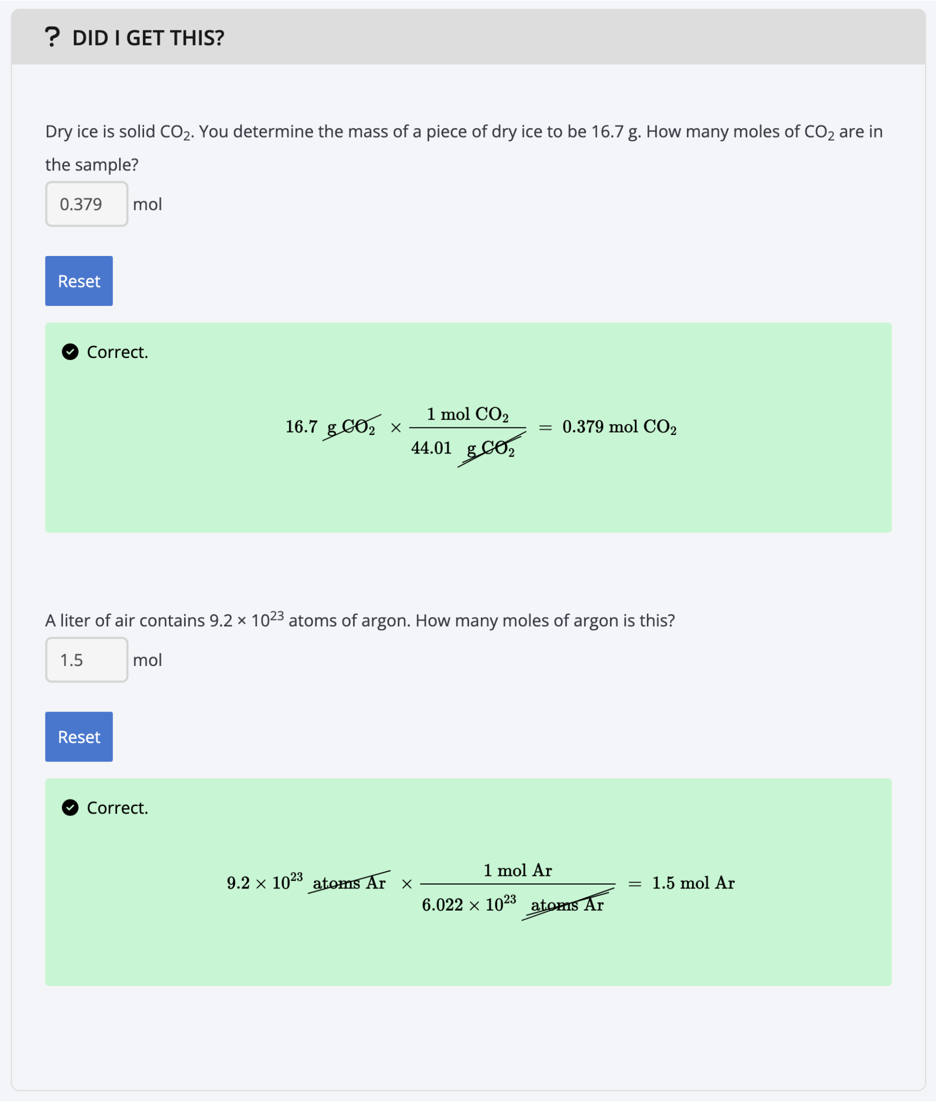

S1.3 Math level 2 (medium math)

Medium math skills typically involve several straightforward mathematical steps. For instance, determining the empirical formula of a compound requires multiple simple conversions between grams and moles (a low math skill), followed by calculating the mole ratio by dividing all mole quantities by the smallest value. This ratio is then simplified to the smallest whole number ratio, which may involve some rounding or approximating, and occasionally converting a decimal to a fraction.

KC: *Determine the empirical formula of a compound (math level = 2)*

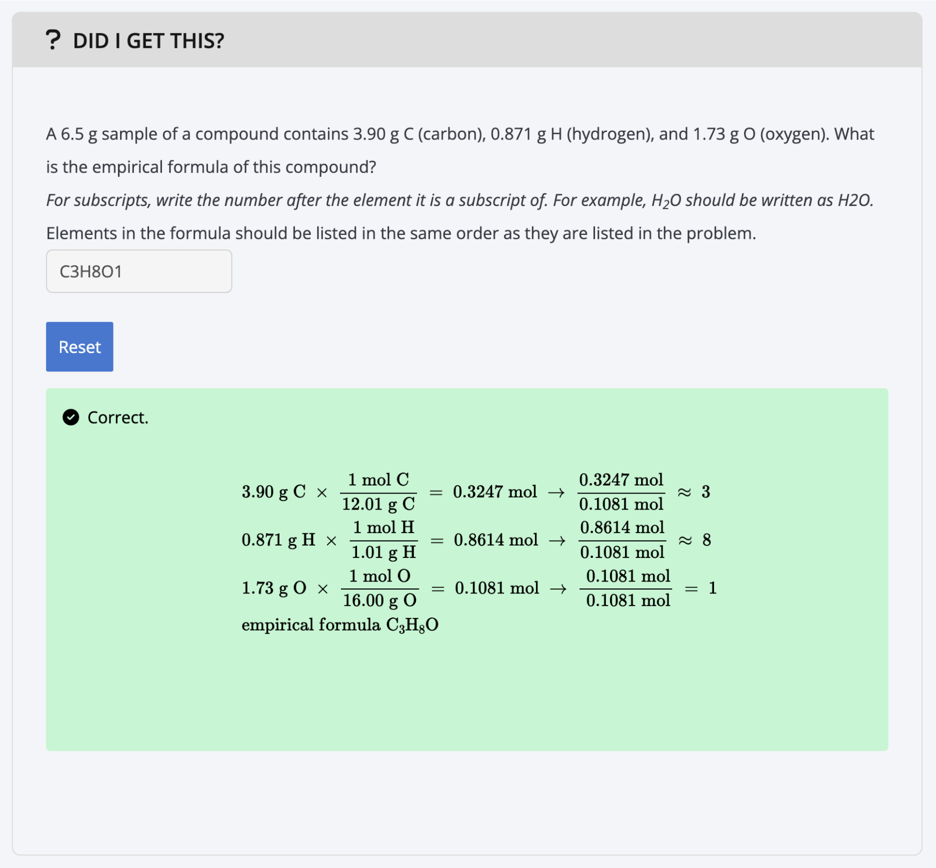

S1.4 Math level 3 (high math)

High math skills involve mathematical demands that exceed those of the lower levels. Notably, there were no skills for which all three coders assigned a high math code (level 3). The highest average rating across all coders was 2.5. In total, 46 out of 348 KCs (13%) have a math level above 2.

KC: *Apply the Clausius-Clapeyron Equation to calculate vapor pressure (math level = 2.33).*

LEARN BY DOING

At 20.0 °C, the vapor pressure of ethanol is 5.95 kPa, and at 63.5 °C, its vapor pressure is 53.3 kPa. Use this information to estimate the enthalpy of vaporization for ethanol.

[ ] kJ/mol

Hints

Temperature must be in kelvin, but pressure can be in any unit as long as both pressure values are the same unit.

1.

$$\Delta H_{vap} = \frac{R \cdot \ln\left(\frac{P_2}{P_1}\right)}{\left(\frac{1}{T_1} - \frac{1}{T_2}\right)}$$

LEARN BY DOING

At 20.0 °C, the vapor pressure of ethanol is 5.95 kPa, and at 63.5 °C, its vapor pressure is 53.3 kPa. Use this information to estimate the enthalpy of vaporization for ethanol.

65 kJ/mol

Reset

Incorrect.

$$\Delta H_{\text{vap}} = \frac{R \cdot \ln\left(\frac{P_2}{P_1}\right)}{\left(\frac{1}{T_1} - \frac{1}{T_2}\right)} = \frac{(8.314\ \text{J/mol}\cdot\text{K}) \cdot \ln\left(\frac{53.3\ \text{kPa}}{5.95\ \text{kPa}}\right)}{\left(\frac{1}{293.15\ \text{K}} - \frac{1}{336.5\ \text{K}}\right)} = 41,483\ \text{J/mol}\ =\ 41.5\ \text{kJ/mol}$$

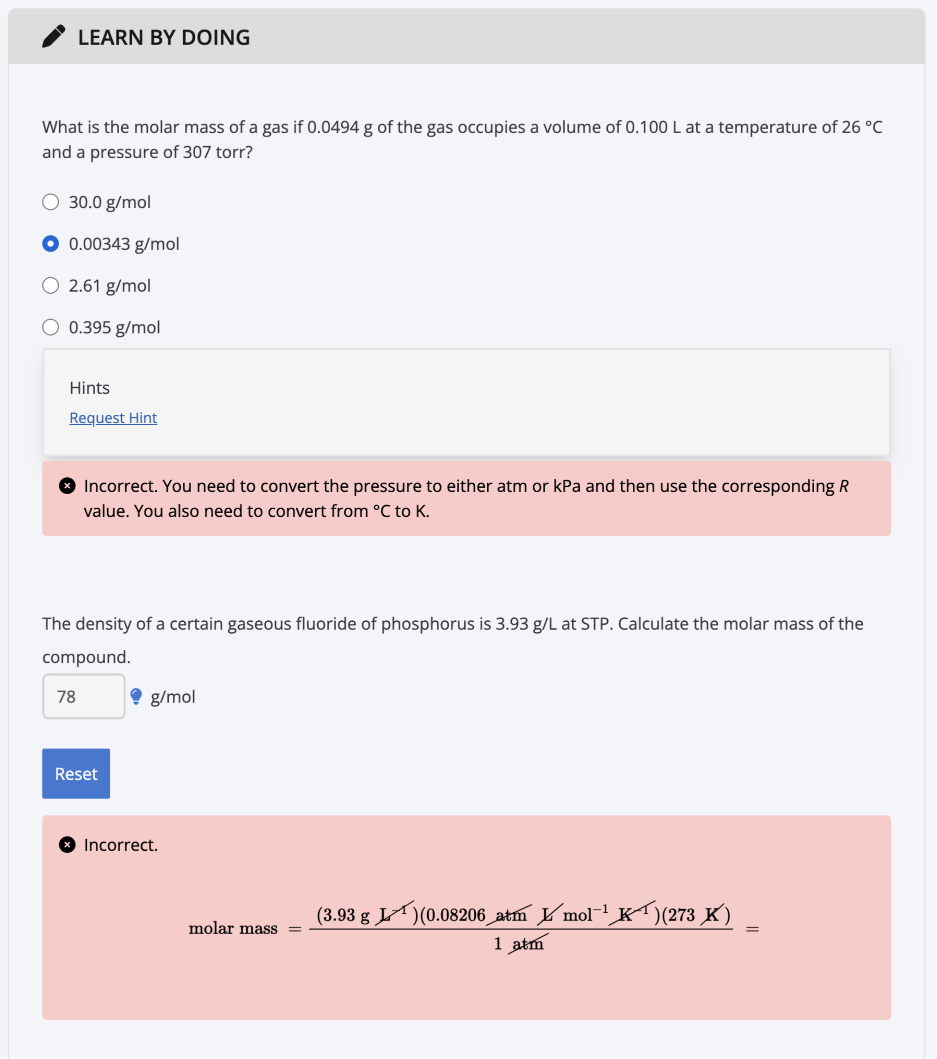

S2 Equations in R Notation for Input to the `glmer` Function

Equations 1 through 3 in the main manuscript define the logistic regression models used to address our three research questions. Below are the corresponding R expressions used to implement these regressions using the `glmer` function.

Equation 1 in R notation:

success $\sim$ opportunity + (opportunity|individual) + (opportunity|KC)

Equation 2 in R notation:

success $\sim$ opportunity $*$ math_level + (opportunity|individual) + (opportunity|KC)

Equation 3 in R notation:

success ~ opportunity + math_level + (0+math_level|individual) + (opportunity|individual) + (opportunity|KC)

S3 Demographic Information

Section 3.1 Data Collection of the main manuscript provides a summary of demographic information self-reported by students. Here, we provide additional details on these survey results.

The demographic information was collected from an optional survey provided within the courseware. Students were given multiple racial categories to select from, and their responses were grouped as follows: participants who selected one or more of *Hispanic*, *Latino*, *Spanish*, *Middle Eastern or North African*, *American Indian or Alaska Native*, and/or *Black or African American* were classified as *underrepresented (UR)*; those who selected one or more of *White* and/or *Asian* were classified as *non-underrepresented (non-UR)*; participants who selected at least one option from both groups were categorized as *mixed.*. *Mixed* is reported separately here, but combined with UR in the main manuscript.

In Study 1, 98 participants (54% of the 183 students included in the study) provided racial identity data, with 48 identifying as UR (49%), 37 as non-UR (38%), and 13 (13%) as mixed. Of the 102 responses (56% of students) to the first-generation college student question, 56 (55%) identified as first-generation. Of the 102 responses (56% of students) to the question on gender, 40 (39%) identifying as male, 56 (55%) as female, and 6 (6%) did not select a male/female gender.

In Study 2, 133 participants (48% of the students included in the study) responded to the racial identity question, with 67 (50%) identifying as UR, 48 (36%) as non-UR, and 18 (14%) as mixed. Of the 135 responses to the first-generation question, 64 (47%) identified as first-generation. Of the 122 responses to the question on gender, 58 (48%) identifying as male and 64 (52%) as female.

S4 Constrasting Study 1 with Study 2

To examine the differences between studies 1 and 2, we extended the regression used for RQ2 (Eq. (2) in the main manuscript), to allow the initial knowledge and learning rate to have a dependence on the study-ID, $S = 0$ for study 1 and $S = 1$ for study 2. The regression formula is:

$$\begin{aligned} P_{i,j} = &\left(\theta + \theta_i + \theta_s S + \sum_k q_{j,k}\left(\beta_k + \boldsymbol{ML_k}\right)\right) \\ &+ \left(\sum_k q_{j,k}(\delta + \delta_i + \delta_s S + \gamma_k + \boldsymbol{NL_k})T_{i,k}\right) \end{aligned} \tag{S1}$$

where θ_s and δ_s are the dependence of the initial knowledge and learning rate on the study-ID, S, and the remaining variables are as for Eq. (3) in the main manuscript.

The data from the two studies was combined and the results of the regression are shown in Table 1. The dependence of initial knowledge on study-ID is not statistically significant ($p = 0.42$). The learning rate does have a significant dependence on study-ID (p = 1.30e-8), with the learning rate being 17% higher for study 2 than study 1.

Table 1: Results of the impact of the study ID on initial knowledge and learning rate from the regression analysis of Eq. S1.

	**Estimate (Std Error)**	**P value**
**Study 1**		
Initial knowledge (θ)	1.169 (0.11)	<2e-16 ***
Learning rate (δ)	0.060 (0.019)	0.00126 **
Initial knowledge by math level (M)	-0.583 (0.075)	7.22e-15 ***
Initial knowledge by study-ID (θ_s)	-0.025 (0.032)	0.42
Learning rate by math level (N)	0.019 (0.014)	0.167
Learning rate by study-ID (δ_s)	0.0103 (0.0018)	1.30e-8 ***

*(Signif. codes: 0 '***' 0.001 '**' 0.01 '*' 0.05 '.' 0.1 ' ' 1)*

S5 Regression Analyses of Math and Chemistry Initial Knowledge Effects on Opportunities

Section 4.3 of the main manuscript contains the following paragraph:

> "In a follow-up regression analysis (see *Supporting Information*) we found that students' chemistry and math initial knowledge both have a significant and independent association with students' total practice opportunities. This result is consistent with the idea that students coming into the course less well-prepared in either math or general chemistry end up pursuing fewer opportunities. This result is perplexing given the high similarity in learning rates: *Lower prior knowledge students pursue fewer opportunities even though they are making progress at about the same rate as students with higher prior chemistry and math preparation."*

The following are the results from this regression.

S5.1 Study 1

Multiple linear regression with *total opportunities* as the dependent variable, and math_intercept (referred to as *Math Initial Knowledge* in the main manuscript) and chem_intercept (referred to as *Chemistry Initial Knowledge* in the main manuscript) as the independent variables.

R Call:

lm(formula = **total_opportunities ~ math_intercept + chem_intercept**,

data = df_student_engagement_ds4856_corr)

Residuals:

Statistic	Min	1Q	Median	3Q	Max
Value	-1562.0	-741.1	-238.5	794.3	2698.8

Coefficients:

**Parameter**	**Estimate**	**Std. Error**	**t value**	**Pr(>|t|)**
(Intercept)	1568.7	316.8	4.952	1.68e-06 ∗
math_intercept	1210.1	446.4	2.711	0.00736 ∗∗
chem_intercept	466.6	148.3	3.148	0.00193 ∗∗

*Signif. codes: 0 '***' 0.001 '**' 0.01 '*' 0.05 '.' 0.1 ' ' 1*

Residual standard error: 998.3 on 180 degrees of freedom

Multiple R-squared: 0.09278, **Adjusted R-squared:** 0.0827

F-statistic: 9.205 on 2 and 180 DF, **p-value:** 0.0001563

Simple linear regression with *total opportunities* as the dependent variable, and math_intercept (referred to as *Math Initial Knowledge* in the main manuscript) as the independent variable.

lm(formula = **total_opportunities ~ math_intercept**, data = df_student_engagement_ds4856_corr)

Residuals:

Statistic	Min	1Q	Median	3Q	Max
Value	-1526.6	-807.6	-198.6	801.0	2625.6

Coefficients:

**Parameter**	**Estimate**	**Std. Error**	**t value**	**Pr(>|t|)**
(Intercept)	2146.0	264.6	8.111	7.39e-14 ∗ ∗ ∗
math_intercept	1298.8	456.3	2.846	0.00493 ∗∗

*Signif. codes: 0 '***' 0.001 '**' 0.01 '*' 0.05 '.' 0.1 ' ' 1*

Residual standard error: 1023 on 181 degrees of freedom

Multiple R-squared: 0.04285, **Adjusted R-squared:** 0.03756

F-statistic: 8.102 on 1 and 181 DF, **p-value:** 0.004931

Simple linear regression with *total opportunities* as the dependent variable, and chem_intercept (referred to as *Chemistry Initial Knowledge* in the main manuscript) as the independent variable.

lm(formula = **total_opportunities** $\sim$ **chem_intercept**, data = df_student_engagement_ds4856_corr)

Residuals:

Statistic	Min	1Q	Median	3Q	Max
Value	-1607.1	-816.6	-303.8	758.2	2870.9

Coefficients:

**Parameter**	**Estimate**	**Std. Error**	**t value**	**Pr(>|t|)**
(Intercept)	867.6	186.1	4.662	6.07e-06 $***$
chem_intercept	492.0	150.5	3.269	0.00129 $**$

*Signif. codes: 0 '***' 0.001 '**' 0.01 '*' 0.05 '.' 0.1 ' ' 1*

Residual standard error: 1016 on 181 degrees of freedom

Multiple R-squared: 0.05574, **Adjusted R-squared:** 0.05053

F-statistic: 10.69 on 1 and 181 DF, **p-value:** 0.001293

S5.2 Study 2

Multiple linear regression with *total opportunities* as the dependent variable, and math_intercept (referred to as *Math Initial Knowledge* in the main manuscript) and chem_intercept (referred to as *Chemistry Initial Knowledge* in the main manuscript) as the independent variables.

R Call:

lm(formula = **total_opportunities** $\sim$ **math_intercept** + **chem_intercept**,

data = df_student_engagement_study2_corr)

Residuals:

Statistic	Min	1Q	Median	3Q	Max
Value	-1377.4	-919.5	-257.5	935.7	2410.3

Coefficients:

**Parameter**	**Estimate**	**Std. Error**	**t value**	**Pr(>|t|)**
(Intercept)	2000.63	299.99	6.669	1.43e-10 $***$
math_intercept	1211.47	420.69	2.880	0.0043 $**$
chem_intercept	-23.16	125.35	-0.185	0.8536

*Signif. codes: 0 '***' 0.001 '**' 0.01 '*' 0.05 '.' 0.1 ' ' 1*

Residual standard error: 1036 on 272 degrees of freedom

Multiple R-squared: 0.02959, **Adjusted R-squared:** 0.02246

F-statistic: 4.147 on 2 and 272 DF, **p-value:** 0.01682

Simple linear regression with *total opportunities* as the dependent variable, and math_intercept (referred to as *Math Initial Knowledge* in the main manuscript) as the independent variable.

lm(formula = **total_opportunities** ∼ **math_intercept**, data = df_student_engagement_study2_corr)

Residuals:

Statistic	Min	1Q	Median	3Q	Max
Value	-1371.3	-926.3	-253.7	909.4	2419.9

Coefficients:

**Parameter**	**Estimate**	**Std. Error**	**t value**	**Pr(>|t|)**
(Intercept)	1969.6	248.2	7.937	5.39e-14 $***$
math_intercept	1205.3	418.6	2.879	0.0043 $**$

*Signif. codes: 0 '***' 0.001 '**' 0.01 '*' 0.05 '.' 0.1 ' ' 1*

Residual standard error: 1034 on 273 degrees of freedom

Multiple R-squared: 0.02947, **Adjusted R-squared:** 0.02592

F-statistic: 8.29 on 1 and 273 DF, **p-value:** 0.004302

Simple linear regression with *total opportunities* as the dependent variable, and chem_intercept (referred to as *Chemistry Initial Knowledge* in the main manuscript) as the independent variable.

lm(formula = **total_opportunities** ∼ **chem_intercept**, data = df_student_engagement_study2_corr)

Residuals:

Statistic	Min	1Q	Median	3Q	Max
Value	-1276	-935	-311	1023	2218

Coefficients:

**Parameter**	**Estimate**	**Std. Error**	**t value**	**Pr(>|t|)**
(Intercept)	1271.512	163.037	7.799	1.33e-13 $***$
chem_intercept	5.498	126.609	0.043	0.965

*Signif. codes: 0 ‘***’ 0.001 ‘**’ 0.01 ‘*’ 0.05 ‘.’ 0.1 ‘ ’ 1*

Residual standard error: 1049 on 273 degrees of freedom

Multiple R-squared: 6.906e-06, **Adjusted R-squared:** -0.003656

F-statistic: 0.001885 on 1 and 273 DF, **p-value:** 0.9654

S6 Additional Results from Study 2

This section shows results for study 2, using formats that are parallel to those used to show results for study 1 in the main manuscript.

Table 2: Distribution of math-level ratings for Study 2, shown as in Table 1 in the main manuscript.

Math rating	0	0.01-1	1.01-2	2.01-3
KCs (%)	33 (10%)	137 (40%)	132 (38%)	46 (13%)
Steps (%)	423 (12%)	1711 (43%)	1522 (39%)	256 (6%)
Opportunities per student KC	4.8	4.0	4.0	2.3
Average performance	0.77	0.71	0.66	0.52
Students	264	270	260	214

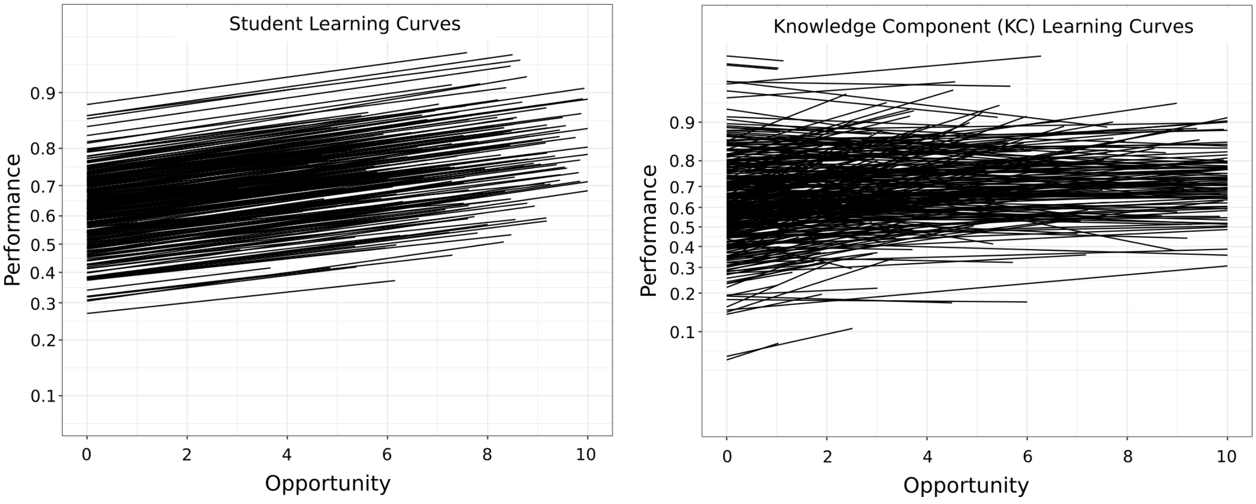

Figure 1: Student learning curves (left panel) and KC learning curves (right panel) for study 2, shown as in Figure 2 of the main manuscript.

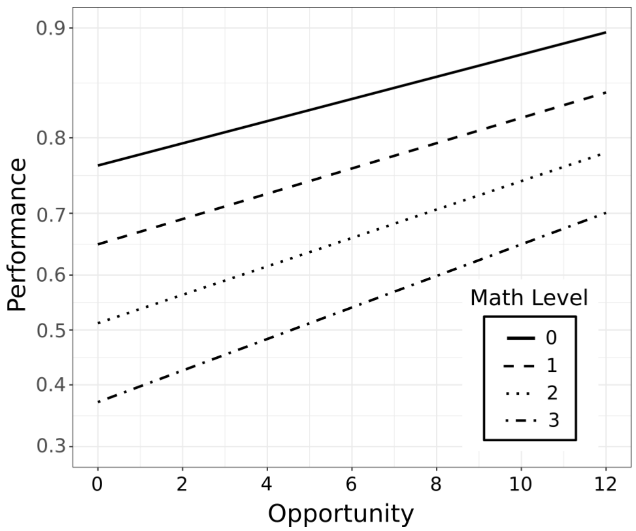

Figure 2: Learning curves generated from the regression parameters for study 2 listed in Table 3 of the main manuscript, shown as in Figure 3 of the main manuscript.

.

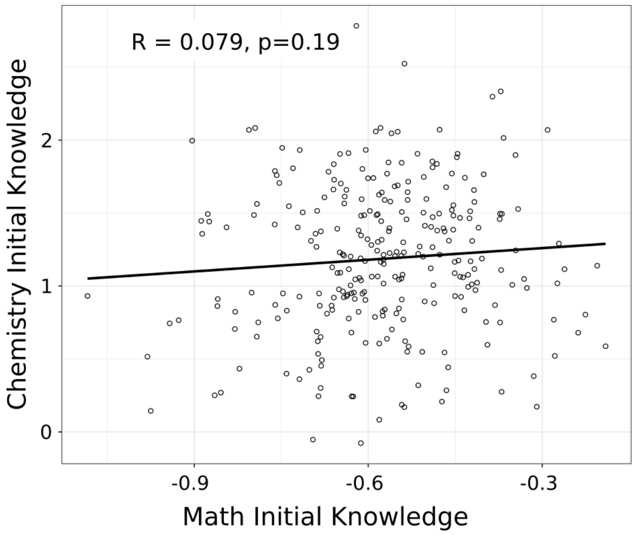

Figure 3: Correlation between students' Math Initial knowledge and Chemistry Initial Knowledge, shown as in Figure 4 of the main manuscript.

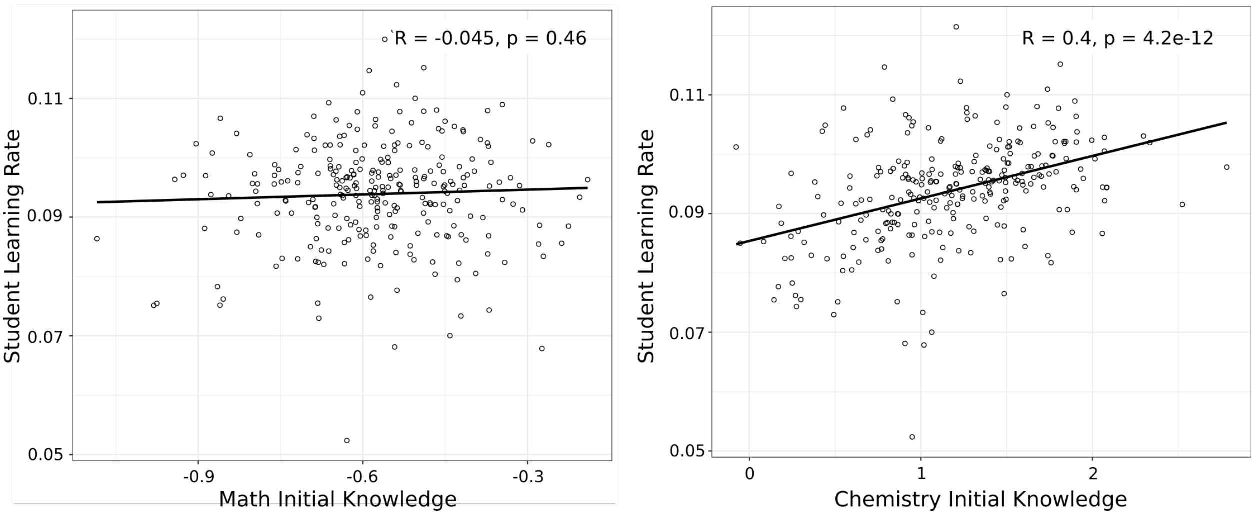

Figure 4: Correlation of students' learning rates with Math and Chemistry Inititial Knowledge, shown as in Figure 5 of the main manuscript.
